# Validation of scrambling methods for vocal affect bursts

**DOI:** 10.3758/s13428-023-02222-1

**Published:** 2023-09-06

**Authors:** Annika Ziereis, Anne Schacht

**Affiliations:** Department for Cognition, Emotion and Behavior, Affective Neuroscience and Psychophysiology Laboratory, Institute of Psychology, University of Göttingen, Göttingen, Germany

**Keywords:** Scrambling, Affect burst, Auditory, Phase, Frequency, Validation, Valence

## Abstract

Studies on perception and cognition require sound methods allowing us to disentangle the basic sensory processing of physical stimulus properties from the cognitive processing of stimulus meaning. Similar to the scrambling of images, the scrambling of auditory signals is aimed at creating stimulus instances that are unrecognizable but have comparable low-level features. In the present study, we generated scrambled stimuli of short vocalizations taken from the Montreal Affective Voices database (Belin et al., Behav Res Methods, 40(2):531–539, 2008) by applying four different scrambling methods (frequency-, phase-, and two time-scrambling transformations). The original stimuli and their scrambled versions were judged by 60 participants for the apparency of a human voice, gender, and valence of the expressions, or, if no human voice was detected, for the valence of the subjective response to the stimulus. The human-likeness ratings were reduced for all scrambled versions relative to the original stimuli, albeit to a lesser extent for phase-scrambled versions of neutral bursts. For phase-scrambled neutral bursts, valence ratings were equivalent to those of the original neutral burst. All other scrambled versions were rated as slightly unpleasant, indicating that they should be used with caution due to their potential aversiveness.

Are emotional stimuli processed differently from non-emotional stimuli? As simple as this question appears, and irrespective of the stimulus modality, it is methodologically challenging to disentangle the impacts of emotional quality from other stimulus properties. For example, an image of a smiling person and an image of the same person with a neutral facial expression differ in terms of emotional valence, but also, to some extent, in their low-level properties. In this example, local differences in low-level properties might even increase with the intensity of the emotional expressions shown; for example, smiling with an open mouth and showing teeth will result in a higher number of bright pixels in the mouth region compared to the corresponding closed mouth or a neutral expression.

In particular, physical stimulus features such as luminance, size, and contrast impact early visual processing (e.g., Bobak et al., 1987; Johannes et al., 1995; Korth & Nguyen, 1997; Marcar & Wolf, 2021), leading to problematic confounds of emotion-related effects with other stimulus effects, which is especially relevant for electrophysiological and imaging research. Importantly, not all stimulus properties are related to the inherent emotional meaning of a stimulus. Thus, to differentiate between emotion-sensitive and emotion-insensitive functional processing units (e.g., single neurons or larger spatial and temporal regions of interest), one would need to keep low-level properties comparable but eliminate those properties related to the emotional valence of an image. For instance, the face-sensitive N170 event-related potential (ERP) component (e.g., Bentin et al., 1996) has been suggested to be already sensitive to emotional expressions (for reviews, see Hinojosa et al., 2015; Schindler & Bublatzky, 2020; but see Rellecke et al., 2012). Whether such early differentiation of a signal is based on a functional detection of an emotional quality or an artifact of confounding low-level features has highly relevant implications for theoretical models of face perception (Bruce & Young, 1986). In the visual domain, a frequently used methodological approach is to compare the processing of intact images with scrambled versions of these images. There are different forms of visual scrambling, for example, shuffling of individual or chunks of pixels (used in, e.g., Cano et al., 2009; George et al., 1996; Herrmann et al., 2004; Latinus & Taylor, 2006; Linkenkaer-Hansen et al., 1998), or shuffling of windows in the frequency or phase domain (used in, e.g., Jacques & Rossion, 2004; Rossion & Caharel, 2011; Schindler et al., 2021), combinations thereof (used in, e.g., Coggan et al., 2017; Sadr & Sinha, 2004), using cyclic wavelet transformations (Koenig-Robert & VanRullen, 2013), or computational models of object recognition (Stojanoski & Cusack, 2014). All methods have in common that they are implemented with the aim of preserving a significant amount of the low-level properties (e.g., luminance, color histograms, frequency spectrum, contrast), while eliminating the identifiability or the semantic properties of a stimulus.

Analogous to the visual domain, the same potential confounds apply in the context of auditory processing. Thus, the investigation of emotional sounds and affective prosody in speech or nonspeech vocalizations requires methods to create non-emotional references with comparable low-level properties (Jürgens et al., 2018; Lausen & Hammerschmidt, 2020). Scrambled versions of auditory stimuli have been implemented in particular to identify voice-sensitive and voice-selective areas in the human auditory cortex (e.g., Belin et al., 2002). Scrambling has also been used to investigate the sensitivity of the amygdala, insula, and superior temporal sulcus to emotional sounds and human vocalizations (Zhao et al., 2016) and in research on music (Menon & Levitin, 2005). Similar to the visual domain, auditory scrambling involves procedures such as time scrambling, i.e., cutting the signal into time bins and shuffling them (used in, e.g., Angulo-Perkins & Concha, 2019; Jiang et al., 2013; Menon & Levitin, 2005; Wilf et al., 2016), phase scrambling (e.g., Gazzola et al., 2006; Zhang et al., 2021), frequency scrambling (Barbero et al., 2021; e.g., Belin et al., 2002), gammatone filter banks (Minagawa-Kawai et al., 2010; Patterson et al., 1995), or combinations of methods (e.g., Coggan et al., 2016; Dormal et al., 2018), preserving different types of low-level features of the stimulus. The question that emerges, therefore, is which method (including specific parameters) is most appropriate.

The aim of the present study was to compare different scrambling methods for creating non-emotional instances of affect bursts (Scherer, 1994), i.e., human nonspeech vocalizations (Schröder, 2003). Moreover, we were interested in how the scrambled versions would be rated in terms of their valence and whether certain levels of stimulus semantics would be preserved, e.g., whether a human voice and/or the speaker’s gender would be recognizable. The two main reasons for this were to (a) create stimuli for experimental tasks (e.g., a gender decision task) and (b) investigate potential valence effects of stimuli as a result of the scrambling procedures.

## Method

### Participants

Data were collected from 62 participants, of which two were excluded from the following analysis because they did not differentiate between the original stimuli. The remaining 60 participants (41 female, 19 male, 0 diverse; *M*_age_ = 29.2 years, range_age_ = [18; 70]) reported normal or corrected-to-normal hearing. Participants were informed of the study procedure and data policy, and informed written consent was obtained. For reimbursement, participants could choose between course credit or a sound file of a scrambled version of their voice.

### Stimuli

Original sound stimuli were short affect bursts, i.e., nonspeech vocalizations, from a validated database (Montreal Affective Voices; Belin et al., 2008). We selected affect bursts from ten different speakers, half of which were female and half male (ID6m, ID42m, ID45f, ID46f, ID53f, ID55m, ID58f, ID59m, ID60f, and ID61m). For each speaker, we included bursts expressing anger, happiness, or a sustained neutral tone. The stimuli varied in duration, ranging from 0.24 to 2.61 seconds.

### Scrambling

**Fig. 1:**
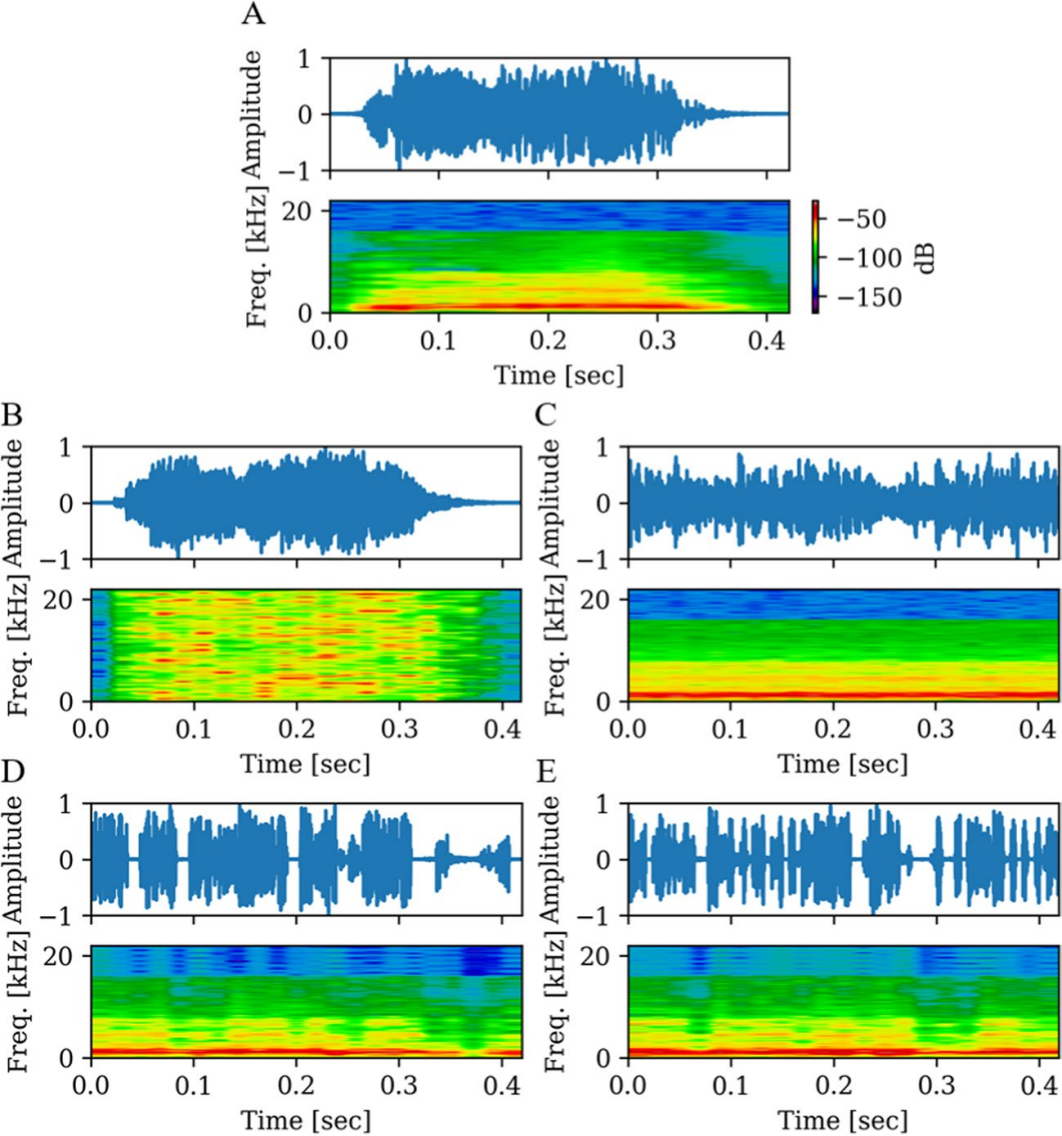
Example of the amplitudes (envelope) and the power spectral density of the original stimulus and scrambled versions. *Notes:* **A** The original, angry bursts stimulus of ID46. **B** Frequency-scrambled version, **C** phase-scrambled version with scrambling frequencies above the median pitch of the stimulus. **D** Time-scrambling of 12-ms windows. **E** Time-scrambling of 6-ms windows. Both time-scrambling versions include amplitude ramps of 1 ms at the beginning and end of each window and thus also differed from the original stimulus in the overall energy

We used four different scrambling methods to manipulate the original stimuli: frequency scrambling, phase scrambling, and two versions of time scrambling. All methods resulted in different acoustic aspects. The amplitude envelope remained similar for frequency sampling, whereas all other methods changed the envelope to a more uniform shape, with more spiky envelopes for the time-scrambled versions. Frequency and phase scrambling preserved the overall energy, which was reduced to some extent for the time scrambling, due to the implementation of amplitude ramps (see below). The Python code for the different scrambling methods and the scrambled versions of the stimuli are available at https://osf.io/uat6m. An exemplary visualization of the sound envelopes and frequency spectra of one original stimulus and its manipulations is shown in Fig. 1.

#### Frequency scrambling

We used an adapted version of the frequency scrambling of Belin et al. (2002). After importing the audio files of the original stimuli and normalizing the amplitudes, we trimmed the array of samples to obtain full-size windows (1024 samples per window) for the Fourier transformation. In incremental steps of 512 samples, we applied the real fast Fourier transformation and shuffled the respective frequencies (by shuffling the positions of the Fourier-transformed values), while keeping the amplitude as in the original window. After applying the inverse fast Fourier transformation, all windows were combined and normalized.

#### Phase scrambling

The phase scrambling was adapted from Gazzola et al. (2006). Instead of using an arbitrary threshold, we used stimulus-specific frequency thresholds to account for gender- and valence-specific differences. The median pitch of each stimulus was extracted using Praat software (Boersma & Weenink, 2018). Based on descriptions of Belin et al. (2008), we used a larger pitch analysis range (75–2000 Hz) for the pitch extraction to account for female and male affective bursts. In contrast to the frequency scrambling method, after Fourier transformation, the frequencies were separated based on the threshold frequency (pitch). We scrambled the phases of the higher frequencies by power-transforming the amplitudes, taking the arc tangent, and shuffling the array. The inverse-transformed array was then merged with the unshuffled values, back-transformed to the time domain, and normalized.

#### Time scrambling

The resolution for temporal differences in human hearing is approximately 4 ms (Samelli & Schochat, 2008). Based on this threshold, we cut the normalized sound files into 6-ms (and 12-ms) windows, shuffled them, and added a 1-ms amplitude ramp at the beginning and end of each bin to eliminate crackling noise between the recomposed windows. The sound files were normalized prior to export.

### Validation study procedure

**Fig. 2:**
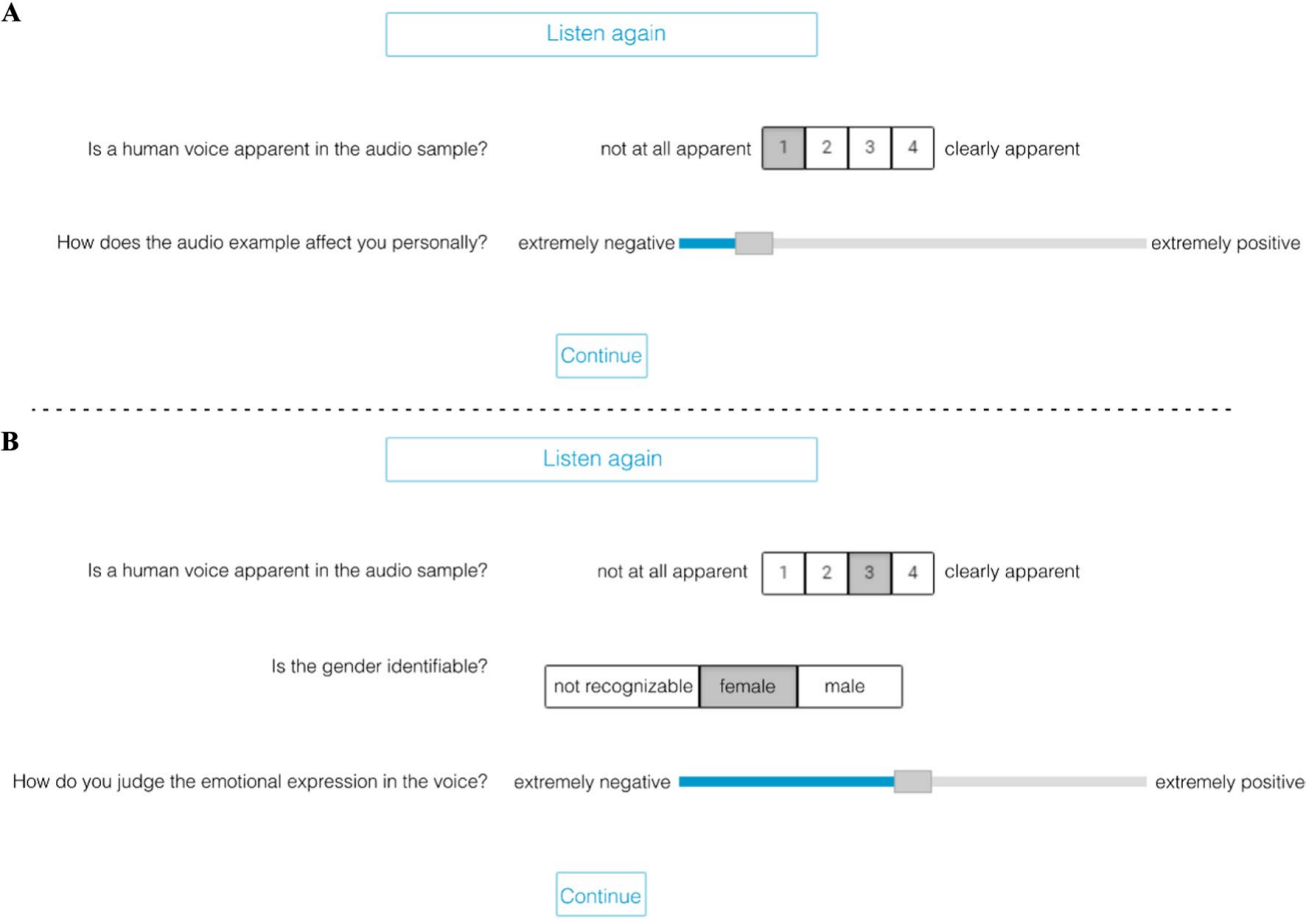
Valence rating procedure. *Notes:* Two example trials of the valence ratings are shown. Auditory stimuli are played automatically at the beginning of a trial. However, participants could listen more often to a stimulus by clicking on “listen again.” The first question was always about the identifiability of a human voice in the stimulus. In **A**, a participant indicated that no human voice was recognizable. Consequently, they rated the stimulus on their subjective reaction. However, in **B**, participants indicated that a human voice was present in the stimulus. In this case, they were asked whether the speaker’s gender was identifiable and how they would judge the valence of the speaker’s expression. Note that the slider poles and appearance were the same for both valence ratings, but the questions differed. After submitting their answers by clicking on “continue,” participants could not go back to previous stimuli

We tested a maximum of ten participants at the same time in a group laboratory. All participants were seated in front of separate test cubicles equipped with headphones (Beyerdynamic DT 770 PRO) and laptops (Dell Latitude E5530 Notebook), all set at a constant, medium volume level. For the stimulus presentation and ratings, we used the survey tool formR (Arslan et al., 2019). After receiving general information about the study and providing written consent and sociodemographic information, participants were presented with an example sound stimulus together with the respective rating scales. Before starting the main validation, open questions about the procedure could be clarified with the experimenter. Participants had to rate the presented sounds along different dimensions. There were a total of 150 stimuli (10 identities, 3 valences, 5 manipulations) to be rated by each participant. The stimuli were presented in randomized order. The questions and rating scales used in the validation study are shown in Fig. 2. Each trial started with an automatic playback of the sound file and the question whether a human voice was apparent in the audio sample, along with a four-point Likert scale, of which the extremes were labeled as “not at all apparent” (1) to “clearly apparent” (4). Depending on the response given in this rating, different follow-up questions were presented. If participants indicated “1” or “2” in the initial rating concerning the presence of a human voice, they were asked, “How does the audio example affect you personally?” (reaction rating) on a slider with labeled poles (left: “extremely negative” and right: “extremely positive”). If participants rated the presence of a human voice with “3” or “4,” they were asked about the speaker’s gender (“not identifiable,” “female,” “male”) and the emotional expression (expression rating) of the voice on a slider with labeled poles identical to the reaction rating (as in Belin et al., 2008). Only the poles of the response sliders were presented without ticks. Internally, values were recorded from 0 to 100 in steps of 1. Participants could listen again to the audio file by clicking on a button presented centrally at the top of the window. This allowed participants who were unsure about what they had heard to re-listen and extract the information they needed to make their decision with greater confidence, similar to giving participants unlimited viewing time to judge a visual stimulus. Before submitting ratings and continuing with the next audio sample, answers could be changed. After responding, it was not possible to return to previous audio samples.

### Statistical analysis

To investigate whether the valence ratings of the scrambled versions were statistically equivalent to the ratings of the original neutral stimuli, we conducted two one-sided tests of equivalence for paired samples. The hypothesis testing for this approach differs from normal paired-sample tests, where researchers aim to test for differences (as opposed to similarities) between two groups. In these cases, the null hypothesis states that the mean of the differences between two samples that are paired is zero. However, if the null hypothesis is not rejected, it is formally incorrect to conclude that there is no effect. Conversely, with large sample sizes, practically irrelevant differences may also be statistically significant. The null hypothesis of equivalence tests for paired samples states that the mean of differences is outside the equivalence interval (−𝛿, 𝛿), of which 𝛿s have to be chosen a priori. When the null hypotheses H_0(1)_: 𝜇_1_ − 𝜇_2_ ≥ 𝛿 and H_0(2)_: 𝜇_1_ − 𝜇_2_ ≤ −𝛿 can be rejected, it can be inferred that the mean of the differences lies within the equivalence interval. We used a nonparametric version of the two-one-sided test of equivalence for paired samples (NPAR, Mara & Cribbie, 2012) due to the non-normality of the voice ratings. As 𝛿 we chose the standard deviation of ratings of the original, neutral burst (𝛿 = 8.63) and compared it to each manipulation and each valence. Nonparametric bootstrapped 95% confidence intervals (*n*_boot_ = 10,000) around the differences were estimated.

## Results

The descriptive results of the valence ratings, voice apparency ratings of the individual stimuli, and gender classification of the stimuli rated as human-like are followed by the model results of the valence-equivalence of the scrambled versions and the original neutral bursts.

### Descriptive results

#### Valence ratings

**Fig. 3:**
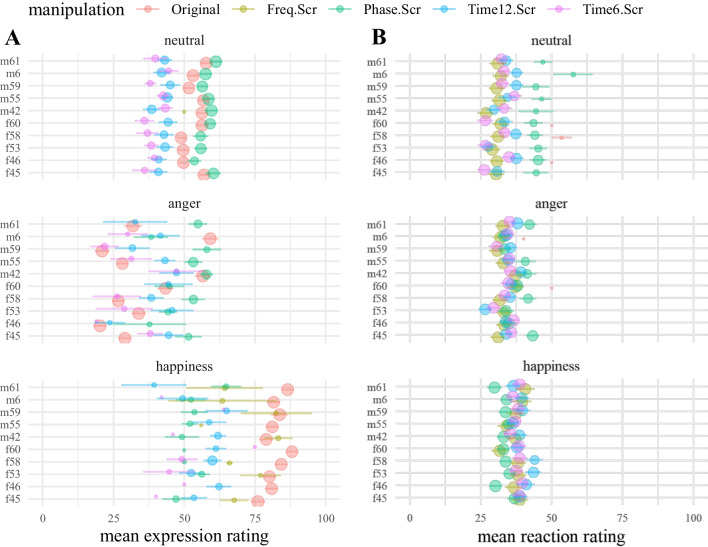
Mean valence ratings by rating type, stimulus ID, valence, and manipulation method. *Notes:* Expression and reaction ratings are shown separately for every speaker ID (*y*-axis) and emotion category. **A** displays mean valence ratings of stimuli in which a human voice was detected. Values represent the rated valence of the speaker’s expression. **B** displays mean valence ratings of stimuli in which no human voice was detected. Here, valence ratings refer to the participants’ reported reaction toward the stimulus. Both sliders’ poles included the labels 0 = “extremely negative” and 100 = “extremely positive.” Error bars show ±1 SE. As unequal numbers of ratings contributed to the valence rating means, we included dot size as a proxy for the number of ratings on which the mean was calculated. Smaller dots indicate fewer ratings, i.e., fewer participants rating the stimulus with regard to the respective rating type (expression vs. reaction)

The type of valence rating depended on whether participants detected a human voice in the sound file. For the original samples, almost all participants detected human voices, whereas for the manipulated stimuli, participants varied in their categorization of human voices, which led to unbalanced group sizes of the ratings and rating types. Figure 3A shows the mean ratings of the speaker’s expression when a human voice was detected. Analogously, Fig. 3B shows the mean ratings of the participant’s reaction to the stimulus when no human voice was detected in the sample.

#### Gender classification and accuracy

If participants indicated the presence of a human voice in a stimulus, they were asked to categorize the speaker’s gender. The accuracy of the gender decision was highest for the original stimuli, although there was some uncertainty for female anger and female neutral stimuli. Although scrambling introduced more uncertainty overall, more correct than incorrect and more correct than uncertain gender ratings were obtained (see Table 1).

**Table 1:** Accuracy of gender ratings of the voices in case a voice was detected

		Original				Phase.scr				Freq.scr				Time12.scr				Time6.scr			
Valence	StimID	Total	Corr.	Wrong	Unsure	Total	Corr.	Wrong	Unsure	Total	Corr.	Wrong	Unsure	Total	Corr.	Wrong	Unsure	Total	Corr.	Wrong	Unsure
Neutral	f45	60	55		5	47	32	2	13					17	13	1	3	9	8		1
	f46	58	53		5	21	13		8					12	9		3	8	7		1
	f53	60	52	2	6	42	30	3	9					26	21	1	4	15	12		3
	f58	57	42	3	12	33	21	1	11					17	16		1	11	9	1	1
	f60	59	51		8	43	25	2	16					21	16		5	14	12		2
	m42	58	58			52	47		5	1			1	24	17	1	6	20	14	3	3
	m55	59	58		1	55	51		4					33	29		4	20	19		1
	m59	59	59			47	46		1					20	20			7	5		2
	m6	60	60			55	53		2					28	26		2	9	8		1
	m61	60	59	1		55	53		2					21	19		2	14	11	1	2
Anger	f45	59	53	1	5	12	5	1	6					12	11		1	7	6		1
	f46	60	49	1	10	4	2		2					3	3			1	1		
	f53	59	47	3	9	8	3		5					7	7			5	4		1
	f58	59	42	8	9	25	8	4	13					13	11		2	4	3		1
	f60	58	34	12	12	5		2	3					5	2	1	2				
	m42	58	58			28	18		10					8	5		3	4	1	2	1
	m55	59	59			32	29		3					10	10			4	4		
	m59	59	59			8	4	1	3					12	8	1	3	12	11		1
	m6	59	59			3	2		1					5	4		1	2	2		
	m61	59	59			20	13		7					3	1		2				
Happi-ness	f45	60	57	1	2	10	5	1	4	3		1	2	7	5		2	1	1		
	f46	59	58		1									11	10		1	1	1		
	f53	58	57	1		8	3	1	4	2			2	14	12		2	3	3		
	f58	59	59			2	1	1		2			2	30	29		1	6	5		1
	f60	59	59			1			1					6	3		3	1	1		
	m42	58	57	1		5	3		2	4			4	13	9		4	1	1		
	m55	59	59			6	3		3	1			1	5	3		2				
	m59	59	56	2	1	5	2		3	2			2	7	2	3	2	1		1	
	m6	59	58		1	5	3		2	4	2		2	7	3		4	1		1	
	m61	59	59			5	2		3	4	1		3	3	2		1				

Counts of correct, wrong, and unsure answers per stimulus ID, valence, and manipulation. Gender classifications were only obtained from participants if they classified a stimulus as entailing a human voice (total). The maximum number of counts equals the number of participants (*N* = 60)

#### Emotional valence of the scrambled affect bursts

To investigate how the scrambled stimuli were perceived in terms of their emotional valence, we decided to collapse the ratings regardless of whether participants rated their emotional reaction to the voice or the valence of the speakers’ expression (see Fig. 2), although we were aware that ratings differed in their meaning. Since the original stimuli were rated almost exclusively on the valence of the speaker’s expression, the participant’s personal reaction cannot be inferred from these types of ratings. The opposite applies to the frequency-scrambled stimuli.

**Fig. 4:**
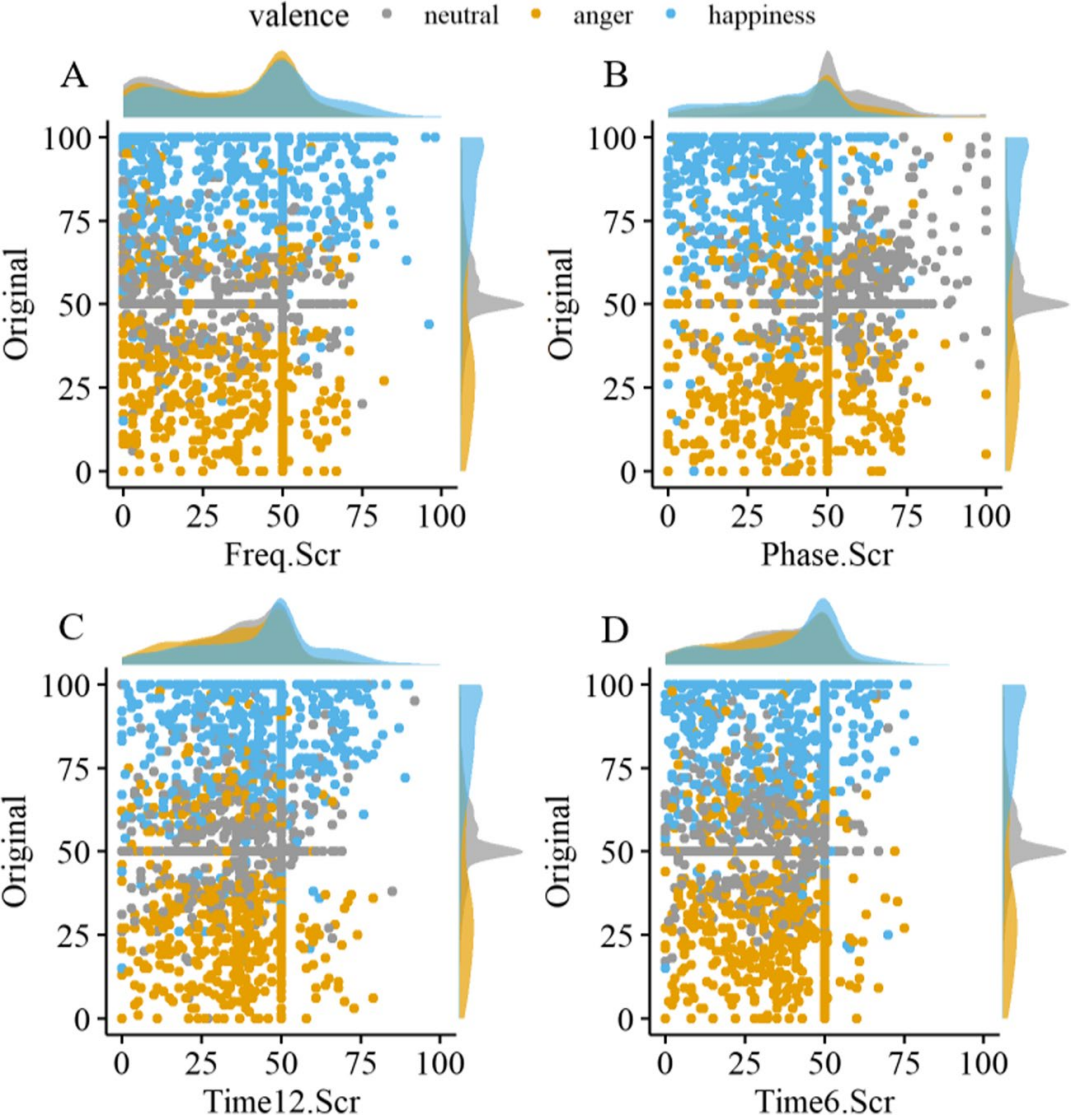
Scatterplots of individual valence ratings of the scrambled vs. the original stimuli. *Notes:* Every dot represents the rating per stimulus ID and participant. On the *x*-axis, the respective manipulated version is plotted against the rating of the unmanipulated, i.e., the original version on the *y*-axis. Panel **A** shows ratings of the frequency-scrambled, **B** of the phase-scrambled, **C** of the 12 ms time-scrambled, and **D** of the 6-ms time-scrambled stimuli. Colors represent the valence category of the original stimulus. Densities of the valence ratings per valence categories are displayed at the top and right sides of the scatterplots

On the one hand, by collapsing the ratings, we chose a relatively liberal criterion for being categorized as neutral, e.g., if participants were uncertain about the valence of the speaker’s expression, they might have been more likely to categorize them as neutral. On the other hand, for a stimulus to be neutral, neither the participant’s reaction to the stimulus nor the speaker’s expression should be identified as very negative or positive. Instead of comparing ratings to a fixed point (e.g., the center of the scale), we compared the ratings of all manipulations and valence categories to the original, neutral stimulus ratings (see Fig. 4).

### Equivalence tests of valence ratings on the scrambled voices

Only phase-scrambled versions of neutral affect bursts were equivalent to the original neutral affect burst ratings (diff = 1, *CI* = [−2.15, 4.35]). No other combination of scrambling method and original valence could be considered equivalent on the basis of the ratings we obtained.^1^ Moreover, the differences were negative across all manipulations and valences, indicating a shift toward negative ratings compared to the original, neutral stimuli. The results of the model are shown in Table 2.

**Table 2:** Results of the two one-sided equivalent tests for the scrambled stimuli

Comparison	Difference from neutral original	*CI*
Freq.Scr neutral	−17.55	[−32.95, −11.25]
Freq.Scr anger	−15.55	[−26.45, −7.40]
Freq.Scr happiness	−6.60	[−17.85, −3.90]
Phase.Scr neutral	1.00	[ −2.15, 4.35]
Phase.Scr anger	−8.20	[−13.60, −5.45]
Phase.Scr happiness	−15.45	[−18.30, −12.75]
Time12.Scr neutral	−14.65	[−18.75, −10.85]
Time12.Scr anger	−16.25	[−18.70, −12.05]
Time12.Scr happiness	−7.05	[ −9.75, −4.35]
Time6.Scr neutral	−18.90	[−21.40, −14.85]
Time6.Scr anger	−16.00	[−18.70, −12.00]
Time6.Scr happiness	−8.75	[−15.75, −5.25]

All stimuli were compared to the ratings of the original version of neutral stimuli. *CI *= 95% nonparametric bootstrapped confidence intervals

## Discussion

The present study compared valence ratings for auditory affect bursts and for different types of their scrambled versions, namely frequency-, phase-, and two time-scrambling approaches, with the aim of creating non-emotional versions of affective stimuli while preserving some of their low-level features. All scrambling approaches reduced the overall valence differences that were present between originally happy, neutral, and angry affect bursts. However, none of the scrambling methods used in this study resulted in neutral-rated versions of the stimuli due to the differential effects of the scrambling methods on the original valence categories.

In addition to the valence ratings, we were interested in whether the stimuli were still perceived as entailing a human voice and gender information depending on the level of distortion of the scrambling methods. Both the judgments of how human-like the stimulus sounded and of the speaker’s gender were affected by the scrambling method and the valence category. Thus, none of the scrambling methods completely preserved the gender information in the stimuli. Phase-scrambled versions of neutral but not happy bursts tended to be classified as entailing a voice compared to not entailing a voice. The rate was also higher for the 12-ms time scrambling than for the 6-ms time scrambling, and overall more pronounced for bursts that were originally of neutral valence. This may have been due to the monotonous melody of neutral bursts, which did not change with the destruction of the temporal coherence. Although the frequency scrambling resulted in the lowest rate of recognizing a human voice in the stimulus descriptively, of these stimuli, the happy frequency-scrambled bursts had the highest rate for recognizing a human voice, probably due to the very characteristic sound envelope of happy bursts (piecewise melody with many brief pauses in between). We observed that in cases where a human voice was detected, gender information was still preserved to some extent, although scrambling increased the perceiver’s uncertainty about the speaker’s gender, as indicated by the accuracy of gender categorizations.

The scrambling methods applied failed to create non-emotional versions of the affect bursts. The clear separation between valence categories observed for the original stimuli was diminished but not completely eliminated for the scrambled versions. The largest difference between valence categories was found for the phase scrambling. Participants’ reaction ratings of phase-scrambled versions of originally neutral stimuli were overall closest to the center of the rating scale, i.e., “neutral,” and thus descriptively more positive than the phase-scrambled versions of happy and angry stimuli (a few participants mentioned that the neutral phase-scrambled stimuli sounded like synthesized sounds of a choir). In particular, phase-scrambled versions of happy bursts were rated descriptively as the most unpleasant of all manipulated happy stimuli. Nevertheless, when testing whether scrambling–valence combinations were equivalent to the original neutral stimulus category, only the phase-scrambled versions of originally neutral stimuli could be considered equivalent in terms of valence ratings. Moreover, other stimulus properties, such as gender information, were detected to a higher degree in phase-scrambled neutral stimuli.

Notably, there was a tendency for scrambled stimuli to be rated as more unpleasant than their original versions. To our knowledge, only a few studies have included explicit valence and arousal ratings of scrambled stimuli. In contrast to our findings, Zhao et al. (2016) presented frequency-scrambled sounds and reported comparable valence and arousal ratings for scrambled and neutral sounds, in addition to scrambled stimuli being rated as meaningless. However, time-scrambled classical music excerpts in Menon and Levitin (2005) were rated both as less pleasant than the original stimuli and as rather unpleasant. To detect potential response tendencies, we investigated the overall rating distributions (Fig. 4) of the original stimuli and the scrambled versions. Several aspects were noteworthy: some asymmetry was observed for the original stimuli, with more extreme (positive) valence ratings for happy stimuli compared to the angry stimuli, and neutral, original stimuli tended to be rated slightly positive. For all scrambling methods and the neutral, original stimuli, there were inflated ratings for the midpoint of the response scale. Due to the nature of the slider responses with initial thumb values, the resolution around the center is low, as participants tend to leave the slider at its default value if it is subjectively close to their (latent) rating. Notably, frequency-scrambled versions appeared to be bimodally distributed, with a second peak at the negative end of the rating scale, i.e., some participants rated them as highly unpleasant.

### Implications

In our study, we used explicit valence ratings. Explicit ratings or categorizations can be viewed as the integrated and cumulative outcome of encoding and appraisal processes and do not necessarily correspond to valence-driven effects at earlier, automatic processing stages (e.g., Hammerschmidt et al., 2017; Rossi et al., 2017; Roux et al., 2010; Walla et al., 2013; Wieser et al., 2006). Thus, our findings do not suggest that scrambled versions of auditory stimuli should not be used in studies of auditory (emotion) processing. However, the assumption of using them as a neutral control may be flawed and may overshadow emotion effects in processing stages that are sensitive to general valence or arousal effects. Moreover, it may be problematic to use scrambled versions as references for difference measures (e.g., negative-scrambled vs. positive-scrambled). In the case of valence differences between scrambled versions, the valence effects of interest might be falsely detected or not detected at all. If the possibility cannot be excluded that the measures of interest are insensitive to valence differences, it might be beneficial to test the homogeneity of scrambled stimulus responses beforehand.

Our findings raised the question of whether there is a fundamental difference between visual and auditory scrambling. Visual scrambling methods have been criticized (e.g., Dakin et al., 2002; Stojanoski & Cusack, 2014), mainly for maintaining or not maintaining important low-level features. However, only a few studies included assessments of valence and arousal for scrambled images, possibly due to the intuitive assumption that without recognizability of affective stimuli, there would be no valence effects (e.g., Braly et al., 2021). Another important aspect is that different low-level features might serve as general valence cues for recognizing emotional stimuli. For example, Delplanque et al. (2007) reported spatial frequencies confounding emotion effects for images selected from the International Affective Picture System (IAPS) database (Lang et al., 2005). Thus, valence effects may resist even in the absence (or reduction) of object recognition and even in the case of earlier processing. For example, arousal and valence of the original stimuli affected mid-latency event-related potentials (ERPs) in their spatially scrambled versions in Rozenkrants et al. (2007). In contrast, no valence effects on mid-latency ERPs of spatially scrambled emotional pictures were reported by Cano et al. (2009).

This study does not come without limitations. Due to the choice of different types of valence ratings (i.e., rating the valence of the expression vs. the subjective reaction to a stimulus), we could not directly compare the ratings of all scrambled versions with the original stimuli. It would have been interesting to test the correlation between the rated valence of the expression and the personal reaction to the original stimuli (one might find laughter highly unpleasant but correctly classify the speaker’s expression as positive). However, by including the expression ratings, we found lower accuracy of the gender classification for some of the original stimuli and more variability in valence ratings for anger bursts. Thus, these stimuli might be problematic for certain experimental tasks. As the age range of our participants was larger than that of the original validation study by Belin et al. (2008), we checked whether the valence effects were related to the age or gender of the participants, which was not the case.

### Outlook

Different stimulus categories may be differently affected by scrambling. Social stimuli such as faces and voices might form special categories due to their high biological relevance (e.g., Belin, 2017) and typicality; for example, faces have been shown to require a higher degree of scrambling before becoming unrecognizable (Stojanoski & Cusack, 2014). There might be a modality-specific divergence of scrambling effects between visual and auditory stimuli. Unlike uncanny-valley effects (for a review, see Kätsyri et al., 2015) for only slight modifications of a facial stimulus (e.g., preserving external facial features but scrambling the eye and/or mouth region), strongly distorted auditory stimuli potentially become more aversive. For example, bursts of white noise are effective and widely used aversive stimuli in fear-conditioning research (Sperl et al., 2016). The specific (nonlinear) function of valence effects of visual and auditory scrambling is an interesting field for future research, especially in the context of research with artificial agents (e.g., Meah & Moore, 2014). A systematic comparison of scrambling methods at different levels of distortion/preservation for social stimuli could help to find adequate comparator stimuli. At the same time, this could provide insight into which low-level properties are relevant cues for social (re)cognition and its sub-domains, including the identification of emotional expression, gender, age, and identity. Candidate sets of acoustic parameters have been identified for affective speech (e.g., Eyben et al., 2010, 2016; Schuller et al., 2009) and nonverbal vocalizations (Sauter et al., 2010). However, testing responses to changing acoustic parameters and their combinations poses challenges for both human participants and machine learning algorithms (e.g., Doğdu et al., 2022) and is likely to be a focus of future research. Another approach to creating neutral versions of stimuli is to synthesize them. Applications could include creating comparable yet novel (emotional or neutral) stimulus instances of the same speaker or expanding stimulus sets to include different speakers. Recent advances in computational speech synthesis, such as speaker-level voice conversion (Walczyna & Piotrowski, 2023) and text-to-speech (TTS) applications, have enabled impressive modifications of speaker-related properties such as gender and identity. Emotion conversion networks that, for example, transform neutrally spoken sentences to sound angry or happy, or vice versa, show promising but still insufficient results (Triantafyllopoulos et al., 2023). Moreover, different classes of emotional auditory stimuli (e.g., affect bursts, interjections, words, sentences with emotional prosody) vary across dimensions and require customized approaches to create neutralized versions while preserving other properties. While the aforementioned tools may work better for affective prosody, they still struggle with affect bursts (but see Baird et al., 2022), possibly due to their greater acoustic variation. Although these tools may at best produce more comparable and standardized stimuli, they would still need to be validated and checked for artifacts caused, for example, by the choice of the training data sets.

## Conclusion

Despite their benefits and intuitive employment as baseline or reference stimuli, scrambled versions of stimuli should be used with caution. The choice of scrambling method should be based on specific hypotheses about which relevant low-level properties should be preserved or eliminated. In the present study, we have shown that in the auditory domain, scrambling methods may interact with the underlying stimulus category, resulting in potentially aversive stimuli. At least for emotion-related research, valence effects of scrambled stimuli should be explicitly tested and controlled for, rather than simply assumed to be “neutral.”

## Data Availability

The raw data of the study are not publicly available for privacy reasons (no consent from participants to publish the raw data) but are available from the corresponding author on reasonable request. Because of this limitation, analysis code is illustrated with an anonymized dataset, which allows readers to check the correctness of their implementation. The study was not preregistered.

## Footnotes

1 To check whether the results might be biased by the older participants’ ratings, we repeated the analysis with a younger subset of participants (*n* = 48, < 35 years, prototypical student sample). There, we found lower valence ratings, especially for the frequency-scrambled versions, compared to the sample including all participants. This difference was less pronounced for the other scrambling methods and may be related to selective hearing loss of higher frequencies in older participants. However, the overall conclusions did not change. That is, in both the total and the younger samples, only the phase-scrambled versions of the neutral original affect bursts were statistically equivalent to the unscrambled original version.
